# Current Understanding of Hearing Loss in Sporadic Vestibular Schwannomas: A Systematic Review

**DOI:** 10.3389/fonc.2021.687201

**Published:** 2021-08-12

**Authors:** Jinlu Gan, Yanling Zhang, Jingnan Wu, Deqiang Lei, Fangcheng Zhang, Hongyang Zhao, Lei Wang

**Affiliations:** ^1^Department of Neurosurgery, Union Hospital, Tongji Medical College, Huazhong University of Science and Technology, Wuhan, China; ^2^Department of Obstetrics and Gynecology, Union Hospital, Tongji Medical College, Huazhong University of Science and Technology, Wuhan, China

**Keywords:** hearing loss, vestibular schwannomas, acoustic neuromas, molecular mechanism, tumor growth pattern, cochlear dysfunction, systematic review

## Abstract

**Objective:**

Hearing loss is the most common initial symptom in patients with sporadic vestibular schwannomas (SVS). Hearing preservation is an important goal of both conservative and surgical therapy. However, the mechanism of SVS-associated hearing loss remains unclear. Thus, we performed this systematic review to summarize the current understanding of hearing loss in the SVS and distill a testable hypothesis to further illuminate its underlying mechanism.

**Methods:**

A systematic review querying four databases (PubMed, Medline, Embase, and Web of Science) was performed to identify studies evaluating hearing loss in patients with SVS and exploring the potential mechanisms of hearing impairment.

**Results:**

A total of 50 articles were eligible and included in this review. After analysis, the retrieved studies could be categorized into four types: (1) 29 studies explore the relationship between hearing loss and the growth pattern of the tumor (e.g., tumor size/volume, growth rate, tumor location, *etc*.); (2) ten studies investigate the potential role of cochlear dysfunction in hearing deterioration, including structural abnormality, protein elevation in perilymph, and cochlear malfunctioning; (3) two studies looked into SVS-induced impairment of auditory pathway and cortex; (4) in the rest nine studies, researchers explored the molecular mechanism underlying hearing loss in SVS, which involves molecular and genetic alterations, inflammatory response, growth factors, and other tumor-associated secretions.

**Conclusions:**

Multiple factors may contribute to the hearing impairment in SVS, including the growth pattern of tumor, cochlear dysfunction, impairment of auditory pathway and cortex, genetic and molecular changes. However, our current understanding is still limited, and future studies are needed to explore this multifactorial hypothesis and dig deeper into its underlying mechanism.

## Introduction

Vestibular schwannomas (VS) are benign tumors originating from Schwann cells of cranial nerve VIII and represent the most common tumors in the cerebellopontine angle (CPA). With the technical advantages in diagnostic techniques and the extension of human life span, the incidence rate of VS has increased steadily from 3 to 34 cases per million every year over the past 40 years (1). Sporadic vestibular schwannomas (SVS) are most common (90%) and develop unilaterally (2). The primary symptoms of SVS are subtle and featured by hearing and balance dysfunction. With occult development, the tumor may affect adjacent nerves, nearby vessels, and brainstem leading to severe neurological manifestations (e.g., facial paralysis, facial numbness, choking, dysphagia, hydrocephalus, *etc*.) (3).

Hearing loss is the initial symptom of SVS and its incidence exceeds more than 90% at the presentation (4). In most patients, the impairment is typically sensorineural in the ear ipsilateral to the tumor, and hearing deteriorates gradually (4). However, in some cases, hearing loss presents in the unaffected ear and worsens suddenly (5, 6). In a recent retrospective study involving 661 SVS patients, the researchers noticed that there might exist a long-term risk of hearing loss in the contralateral ear (5). After surgical and radiation therapy, hearing dysfunction is more prevalent. Hearing preservation, especially in small and medium size tumors, is one of the primary targets in the SVS treatment (7, 8).

Generally, the etiology of hearing loss in SVS can be mainly classified as iatrogenic and tumorigenic. The iatrogenic hearing impairment is directly associated with adopted surgery and radiotherapy. Regarding tumorigenic hearing loss, the underlying mechanism is still unclear. It has been reported that hearing damage was related to multiple factors, including the growth pattern of tumors (e.g., size, growth rate, location, *etc*.), pathological alteration in the inner ear, aberrant inflammatory response, gene mutation, aberrant DNA methylation, etc. (9–13). But the existing data remains controversial. In this review, we focused on the tumorigenic hearing loss in SVS patients and systematically reviewed the literature in the past 20 years to have a landscape view in its current understanding and distill a testable hypothesis to further illuminate its mechanism.

## Methods

A systematic search of the published literature was conducted in the Medline, PubMed, Web of Science, and Embase databases for articles regarding hearing loss in SVS between January 2000 and Dec 2020. And a comprehensive search term was developed using Boolean keywords: “acoustic neuroma(s)”, “vestibular schwannoma(s)”, “hearing loss”, “hearing impairment”, “hypoacusis” and “transitory deafness”. After removing the duplicates *via* Endnote X9 software (Clarivate Analytics^®^), two reviewers independently evaluated these references *via* the titles, abstracts, and full-texts to screen for eligible reports based on predetermined inclusion and exclusion criteria.

To rule out the confounding variables from surgery and radiotherapy, the included clinical study must contain either wait-and-scan or presurgical data. The other inclusion criteria involve: 1) the original reports on the hearing loss in SVS; 2) the studies published between Jan 2000 and Dec 2020; 3) English literature. The exclusion criteria involve 1) case reports or series with the number of patients less than ten; 2) other types of the literature including reviews, book chapters, conference abstracts, editorial notes, letters, etc.; 3) the studies reporting neurofibromatosis type 2, bilateral VS and neoplasms other than VS; 4) the studies investigating the hearing level after surgery and radiation; 5) the studies in which the treatment outcome is served as main findings; 6) unavailable full-texts and non-English literature. If any disagreement occurred about the eligibility of the literature, it would be resolved by consensus after thoroughly discussing with a third researcher.

## Results

**Figure 1:**
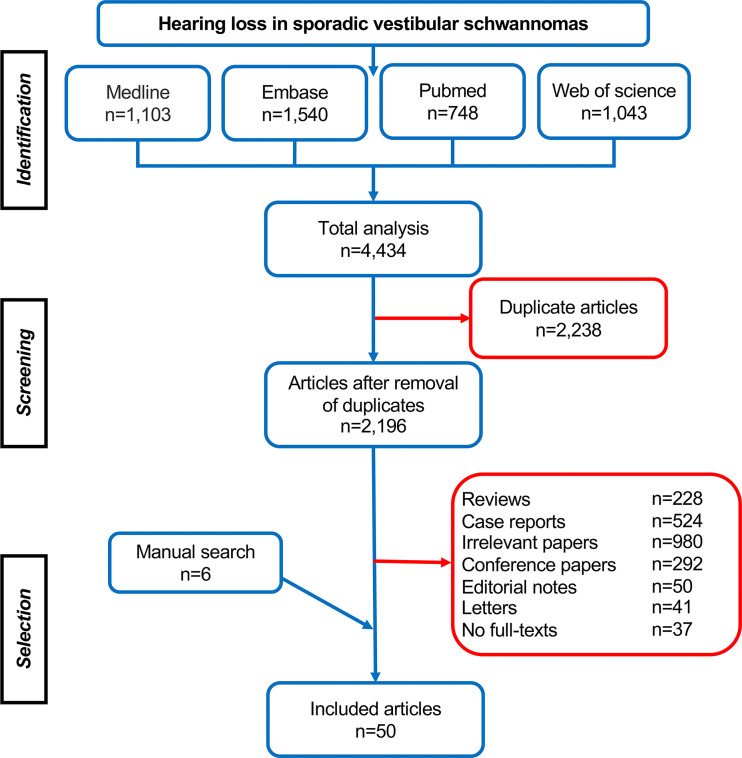
Study selection and characteristics.

In total, 4,434 articles were retrieved, including 1,103 studies from Medline, 1,540 studies from Embase, 748 studies from Pubmed, and 1,043 studies from Web of Science. After removing 2,238 duplicates, 2,196 studies were identified for further selection *via* their titles, abstracts, and full-texts, and the references list of eligible studies was reviewed subsequently for additional studies. Finally, 50 studies were selected according to the inclusion and exclusion criteria and were involved in the systematic analysis (**Figure 1**). Among them, 29 studies investigated the relation of tumor growth pattern (e.g., size, anatomical location, growth rate, *etc*., **Tables 1**–**3**) with hearing, and ten studies focused on cochlear dysfunction (**Table 4**), and two studies looked into the impairment of auditory pathway and cortex, with the rest nine papers exploring its molecular and genetic changes (**Table 5**).

**Table 1:** The summarization of literature regarding the association between tumor size/volume of SVS and hearing loss.

Authors year	Country	No. Patients (M/F)	Tumor size/volume	Main outcomes
**One dimensional measurement/KOOS stage**				
Day, A. S. 2008 (14)	Taiwan	44 (22/22)	Small size (<1.0 cm), medium size (1.0-2.5 cm), large size (>2.5 cm)	A trend of correlation between tumor size and audiographic configuration, with small-sized (<1 cm) tumor in normal and rising types, medium-sized (1.0-2.5 cm) tumor in mid- and high-frequency hearing loss, and large-sized (>2.5 cm) tumor in flat and deafness types.
Tringali, S. 2008 (15)	France	734 (319/415)	KOOS stage T1-4	Stage T4 hearing loss was greater at 250 and 500 Hz and smaller at 2,000 and 8,000 Hz. But there was no difference in the loss of PTA. Additionally, SDS was smaller in Stage T4.
Sakamoto, T. 2001 (16)	Japan	31 (9/22)	Mean tumor size 16.9 mm	No correlation was found between tumor volume and annual hearing loss speed.
Caye-Thomasen, P. 2007 (17)	Denmark	156 (95/61)	IAC tumor size (<0.5 cm, 0.6-1.0 cm, >1.0 cm)	The hearing loss at diagnosis and during observation was not related diagnostic tumor size, tumor induced expansion of the internal auditory canal or tumor sublocation (fundus, central or porus).
Tutar, H. 2013 (18)	Turkey	76 (43/33)	Small (<20 mm) and large tumor (>20 mm)	No correlation was found between the extension of tumor to the IAC, tumor size and hearing loss.
Fayad, J. N. 2014 (19)	USA	114 (57/57)	Mean tumor size 10.5 mm	There was no correlation between the amount of change in hearing and the size of the tumor.
Teggi, R. 2014 (20)	Italy	64 (22/42)	KOSS stage T1-4	Intracanalicular diameter, intracanalicular length and tumor size did not correlate with PTA.
Lee, S. H. 2015 (21)	Korea	114 (46/68)	IAC and extrameatal tumor (<1.0 cm, 1.1-2.5 cm, 2.6-4.0 cm, >4.1 cm)	Audiometry results did not correlate with tumor size.
Cazzador, D. 2017 (22)	Italy	81 (41/40)	Mean tumor size 6.7 mm	In small SVS, hearing status at baseline did not correlate with the initial site and tumor size.
West, N. 2018 (23)	Denmark	124 (58/66)	Extrameatal tumors (<1.0 cm, 1.1-2.0 cm, 2.1-3.0 cm, 3.1-4.0 cm, >4.0 cm)	Increasing tumor size is not directly associated with hearing loss.
**Three-dimensional measurement**				
Gerganov, V. 2009 (9)	Germany	99 (48/51)	KOOS stage T1-4	Tumor volume, tumor stage, coronal diameter, and the distance between the lateral tumor end and the fundus correlated significantly with hearing functions.
Bathla, G. 2016 (24	USA	41 (15/26)	Mean tumor volume 5.5 ml	Maximal anteroposterior and mediolateral dimensions correlated with hearing loss. Total tumor volume calculated *via* 3D volumetric and ABC/2 methods correlated with hearing loss.
Joo, J. 2017 (25)	Korea	97 (37/60)	Mean tumor volume 1.14 ml	Hearing impairment was related significantly to the initial tumor volume (≥0.1 ml).
Patel, N.S. 2020 (26)	USA	213 (91/122)	Median tumor volume 0.12 ml	Larger initial tumor volume was associated with poorer hearing at baseline and it was also associated with the development of non-serviceable hearing during observation.

The color code: green, the study reports a significant correlation between hearing loss and tumor size/volume; yellow, the study reports a trend correlation between hearing loss and tumor size/volume; red, the study reports no correlation between hearing loss and tumor size/volume. 3D, three dimensional; IAC, internal auditory canal; SDS, speech discrimination score; PTA, pure tone average; SVS, sporadic vestibular schwannoma.

**Table 2:** The summarization of literature regarding the association between tumor growth rate and hearing loss.

Authors year	Country	No. Patients (M/F)	Follow-up* (months)	Tumor size/volume*	Main outcomes
**Tumor growth assessed by growth rate (mm/year)**					
Hajioff, D. 2008 (27)	UK	72 (32/40)	121 (80-271 m)	9.8 (3-24.4 mm)	Hearing deteriorated in the stable tumors but did much faster in the growing tumors (>1 mm/year)
Joo, J. 2017 (25)	Korea	97 (37/60)	47 (13-122 m)	Mean tumor volume 1.14 ± 2.89ml	Hearing impairment was related significantly to tumor growth (≥0.10 ml/year).
Sakamoto, T. 2001 (16)	Japan	31 (9/22)	33 (6-92 m)	16.9 (3.0-28.8 mm)	The association between the annual hearing loss and annual tumor growth rate was recognized. However, they were not correlated with PTA at the initial diagnosis.
Prasad, S. C. 2018 (28)	Italy	154 (N.A.)	78 ± 30 m	KOOS stage T1-2	The growing tumors (> 1 mm/year) tended to cause progressive hearing loss, but this was not statistically significant.
Younes, E. 2017 (29)	Lebanon	53 (25/28)	32 (12-60 m)	IAC SVS: 6.2 mm	IAC SVS evolution (>2 mm/year) was not correlated with hearing deterioration with time.
**Tumor growth assessed by the change of tumor size (mm)**					
Walsh, R. M. 2000 (30)	Canada	25 (10/15)	44 (12-194 m)	8.5 ± 3.7 mm	There is a significant risk of hearing loss in the growth tumors (> 1 mm).
Fayad, J. N. 2014 (19)	USA	114 (57/57)	77 ± 61 m	10.5 (2-28 mm)	PTA declined more in the growing tumors.
Van Linge, A. 2016 (31)	Netherlands	155 (80/75)	40 (9-140 m)	IAC and CPA tumors	Hearing loss is associated with tumor growth in intracanalicular tumors.
Kirchmann, M. 2017 (10)	Denmark	156 (95/61)	114(12-300 m)	IAC tumors at the diagnosis	The PTA deterioration in the growing tumors was significantly higher, whereas the rate of SDS decrease was not significant. There was no significant difference in hearing loss progression between tumors with intrameatal growth only and tumors with extrameatal growth.
Caye-Thomasen, P. 2007 (17)	Denmark	156 (95/61)	55 m	IAC tumors (<0.5 cm, 0.6-1.0 cm, >1.0 cm)	The difference in hearing deterioration was not significant between the stable and growing tumor. Correlation analyses showed that the PTA deterioration rate did indeed correlate positively with the absolute growth rate.
Pennings, R. J. 2011 (32)	Canada	47 (19/28)	43 (8-84 m)	IAC tumors	Hearing deterioration occurs in some intracanalicular SVS, regardless of tumor growth.
van de Langenberg, R. 2011 (33)	Netherlands	36 (17/19)	20 (12-67 m)	0.33 (0.05-1.64 ml)	No significant correlation was found between increase in volume and change in hearing function.
Patel, N.S. 2020 (26)	USA	213 (91/122)	36 m	Median tumor volume 0.12 ml	The patients with tumor growth were not significantly more likely to develop non-serviceable hearing during the observation.
**No growing tumors**					
Graamans, K. 2003 (34)	Netherlands	49 (24/25)	84 (12-168 m)	IAC: 9.8 (3-16 mm) CPA: 11.1 (6-20 mm)	Hearing deterioration presents in non-growing SVS.
Patel, N. B. 2015 (35)	USA	15 (4/11)	12-72 m	IAC: 3-14 mm. CPA: 3-15 mm.	Hearing decline is exaggerated in the affected ear despite no vestibular schwannoma growth.

*The item is expressed as median/mean (range) or mean ± standard deviation.

The color code: green, the study reports a significant correlation between hearing loss and tumor growth; yellow, the study reports a trend or ambiguous correlation between hearing loss and tumor growth; red, the study reports no correlation between hearing loss and tumor growth. CPA, cerebellopontine angle; IAC, internal auditory canal; PTA, pure tone average; SDS, speech discrimination score; SVS, sporadic vestibular schwannomas.

**Table 3:** The summarization of literature regarding the association between SVS location and hearing loss.

Authors year	Country	No. Patients (M/F)	Follow-up* (months)	Tumor size*	Main outcomes
**IAC tumors**					
Caye-Thomasen, P. 2007 (17)	Denmark	156 (95/61)	55 m	IAC tumors (<0.5 cm, 0.6-1.0 cm, >1.0 cm)	The hearing loss at diagnosis and during observation was unrelated to tumor sublocalization (fundus, central or porus).
Pennings, R. J. 2011 (32)	Canada	47 (19/28)	43 (8-84 m)	IAC tumors	There were no significant differences in hearing loss by subsite in the internal auditory canal (porus, fundus, central).
Koen, N. 2020 (36)	USA	38 (18/20)	40 ± 32 m	< 5 mm	The findings indicated independence between tumor location and hearing outcomes.
**CPA *vs*. IAC tumors**					
Hajioff, D. 2008 (27)	UK	72 (32/40)	121 (80–271 m)	9.8 (3-24.4 mm)	Hearing deterioration was more significant in CPA tumors than in IAC tumors.
van Linge, A. 2016 (31)	Netherlands	155 (80/75)	39.6 (9-140 m)	IAC and CPA tumors	Patients with IAC tumors presented with lower PTA in comparison with the tumors extending into the CPA.
Lee, S. H. 2015 (21)	Korea	114 (46/68)	N.A.	KOOS stage T1-4	Degree of hearing loss, SDS, tinnitogram findings, and ABR results were not associated with tumor site.
Cazzador, D. 2017 (22)	Italy	81 (41/40)	27.0 ± 17.2 m	6.7 ± 2.9 mm	Hearing status at baseline showed no correlation with the initial location of the SVS.

*The item is expressed as median/mean (range) or mean ± standard deviation.

The color code: green, the study reports a significant correlation between hearing loss and tumor location; yellow, the study reports a trend correlation between hearing loss and tumor location; red, the study reports no correlation between hearing loss and tumor location. ABR, auditory brainstem response; CPA, cerebellopontine angle; IAC, internal auditory canal; N.A., not available; PTA, pure tone average; SDS, speech discrimination score; SVS, sporadic vestibular schwannomas.

**Table 4:** The summarization of literature regarding cochlear dysfunction in SVS-associated hearing loss.

Authors	Year	Country	No. Patients	Main outcomes
**Structural abnormality of cochlea**				
Mahmud, M. R. (13)	2003	USA	11	SVS appeared to cause hearing loss by inducing degenerative changes in the inner ear.
Roosli, C. (37)	2012	USA	32	There was significant degeneration of cochlear structures in affected ears with SVS.
Eliezer, M. (38)	2019	France	23	The volume of the utricle in patients with obstructive SVS moderately correlated with the degree of hearing loss.
Karch-Georges, A. (39)	2019	France	183	Saccular dilation, an MR sign of endolymphatic hydrops, was correlated to hearing loss.
**The change of perilymph**				
Yamazaki, M. (40)	2009	Japan	28	A weak but positive correlation was observed between post-contrast cochlear signal intensity on 3D-FLAIR images and the degree of hearing impairment.
Lee, I. H. (41)	2010	Korea	34	There was no significant correlation between the signal intensity ratios of the labyrinth and the degree of hearing loss.
Kim, D. Y. (42)	2014	Korea	102	The relative signal intensity of the cochlea to the corresponding brainstem correlated with the audiometric findings in patients with IAC SVS but not in patients with CPA SVS.
**Functional alteration**				
Gouveris, H. T. (43)	2007	Germany	39	Amplitudes of the DPOAEs began to decrease even at the early stages of hearing loss in SVS patients, which suggested a cochlear origin of early HL in these patients
Ferri, G. G. (44)	2009	Italy	183	The results confirmed that sensorineural hearing loss due to SVS could be of sensory and neural origin. DPOAEs remained just a complementary auditory test.
Byun, H. (45)	2019	Korea	23	Cochlear DRs were detected in hearing losses associated with unilateral SVS using the TEN tests.

CPA, Cerebellopontine angle; DPOAEs, distortion products of otoacoustic emissions; DRs, dead regions; HL, hearing loss; IAC, intracanalicular or internal auditory canal; SVS, sporadic vestibular schwannomas; TEN tests, the threshold-equalizing noise test.

**Table 5:** The summarization of literature regarding the molecular and genetic change in SVS-associated hearing loss.

Authors	Year	Country	No. Patients	Main outcomes
**NF2 and other genetic alterations**				
Lassaletta, L. (46)	2006	Spain	22	Aberrant methylation of tumor-related genes might contribute to SVS development and TP73 methylation was associated with hearing loss.
Lassaletta, L. (47)	2007	Spain	21	Patients with negative cyclin D1 expression had longer duration of deafness (p = 0.02) and higher 2,000-Hz hearing thresholds (p = 0.04) than cyclin D1+ patients.
Stankovic, K. M. (48)	2009	USA	13	Four genes (PEX5L, RAD54B, PSMAL, and CEA) were possible determinants of HL associated with SVS, and PEX5L, RAD54B, and PSMAL had low expression and CEA was overexpressed in SVS patients with poor hearing.
Lassaletta, L. (11)	2013	Spain	51	Patients with NF2 mutations had lower PTA thresholds compared with those without NF2 mutations.
**Inflammation**				
Dilwali, S. (49)	2015	USA	13	Secreted factors from SVS caused cochlear damage. TNFα was identified as an ototoxic molecule but FGF2 as an otoprotective molecule in SVS secretions.
Sagers, J. E. (12)	2019	USA	30	NLRP3 inflammasome with IL-1ß was preferentially associated with poor hearing in SVS patients.
**Growth factors and other secreted factors**				
Dilwali, S. (50)	2013	USA	35	Secretion of FGF2 was higher in good hearing *versus* poor hearing of SVS based on cytokine array analysis. FGF2 might be otoprotective in SVS.
Dilwali, S. (49)	2015	USA	13	Secreted factors from SVS caused cochlear damage. TNFα was identified as an ototoxic molecule but FGF2 as an otoprotective molecule in SVS secretions.
Soares, V. Y. (51)	2016	USA	6	Human SVS cells from patients with poor hearing produced extracellular vehicles that could damage cultured murine cochlear sensory cells and neurons.
Ren, Y. (52)	2020	USA	23	The expression and activity of MMP-14 in the plasma and tumor secretions correlated with the degree of hearing loss in SVS patients. MMP-14 at physiologic concentrations impaired spiral ganglion neuronal fibers and synapses in cochlear explant cultures.

CEA, carcinoembryonic antigen; FGF2, fibroblast growth factor 2; HL, hearing loss; IL-1ß, interleukin-1ß; MMP 14, matrix metalloprotease 14; NLRP3, NLR family pyrin domain containing 3; PEX5L, peroxisomal biogenesis factor 5-like; PSMAL, prostate-specific membrane antigen-like; PTA, pure tone average; RAD54B, RAD54 homolog B; SVS, sporadic vestibular schwannoma; TNFα, tumor necrosis factor alpha.

## Discussion

The incidence of SVS increases dramatically in recent years because of the advantages of modern imaging examination, especially magnetic resonance imaging (MRI). Hearing loss is present in more than 90% of patients, but its underlying mechanism remains unclear. In this study, we systematically searched the literature on this topic in the past 20 years, and 50 studies were eligibly included. Based on our primary findings, the retrieved studies could be categorized into four types: (1) the studies explored the relationship between hearing loss and the growth pattern of the tumor (e.g., tumor volume, growth rate, tumor location, *etc*.); (2) the studies looked into the pathological changes of the inner ear (e.g., structural abnormality, elevated protein levels in perilymph, functional alterations, *etc*.) and their potential association with hearing loss; (3) the studies evaluated tumor-induced impairment of auditory pathway and cortex, which might lead to progressive hearing deterioration; (4) the studies investigated the underlying molecular and genetic mechanism of SVS-associated hearing loss.

### Tumor Growth Pattern

In theory, hearing loss in SVS may be caused by dysfunction of the cochlear nerve. This retrocochlear mechanism was supported by multiple levels of evidence, including the retrocochlear changes on the measurement of auditory brainstem response (ABR), pathologic data demonstrating atrophy and destruction of the cochlear nerve, morphological and functional alterations in the auditory pathway, *etc*. Among these, the growth pattern of tumors, including tumor size/volume, tumor growth, anatomical location, pressure and tumor filling within the internal auditory canal (IAC), *etc*., serves as the primary focus in the studies regarding hearing loss in SVS. In this review, we retrieved 29 studies on this topic. Among them, 14 papers reported the association between tumor size/volume and hearing loss (**Table 1**), 15 papers focused on tumor growth (**Table 2**), seven papers looked into the anatomical location of the tumor (**Table 3**) with another four papers regarding the pressure and tumor filling within IAC. However, the current reports are conflicting and the association between hearing loss and the SVS growth pattern is unclear.

#### Tumor Size/Volume

The hypothesized pathogenesis of hearing loss in SVS involves mechanical compression of cochlear nerves and brainstem. Intuitively, the larger tumor may lead to severer compression, which will affect their blood supply and lead to neurosensory hearing deficits by subsequent ischemic changes. However, in this systematic review, the majority of the retrieved studies (8/14, 57.1%) suggest the degree of hearing loss do not correlate with tumor size/volume (16–23). At the beginning of this millennium, Sakamoto et al. found no correlation between hearing loss speed and tumor size at the initial diagnosis (16). However, the sample size of this study was small and it involved only 31 patients. The researchers also admitted that it was difficult to estimate tumor diameter in relation to the hearing loss speed because the tumor size of SVS varied along with their observation in each individual (16). Similarly, in a later study involving 156 participants, the researchers looked into the small intracanalicular SVS (the mean largest intrameatal diameter ~6.0 mm). They concluded that the hearing loss at diagnosis and during the observation was not related to tumor size (17). Based on the maximum extrameatal diameter in the axial plane of MRI, Tutar et al. distinguished two SVS groups by maximal diameter with one group <20 mm and the other one >20 mm, and they found that there was no significant correlation between the tumor size and the hearing levels in the pure tone audiometry (PTA), speech discrimination scores (SDS), and speech reception thresholds (SRT) (18). Additionally, Lee et al. and West et al. further grouped SVS according to the extrameatal diameter (from small SVS <1 cm to giant tumor >4 cm) and found no significant correlation between hearing function and tumor grade (21, 23).

In contrast, six studies supported the correlation between hearing loss and tumor size/volume (9, 14, 15, 24–26). In a pilot study with a small sample size (n = 44), Day et al. reported a trend correlation between tumor size and audiometric configuration with small tumor (<1 cm) in normal and rising types, medium tumor (1.0-2.5 cm) in mid- and high-frequency hearing loss, and large tumor (>2.5 cm) in flat and deafness types (14). At the same time, Tringali et al. performed a large prospective study involving 734 patients with KOOS stage T1-4 SVS and observed that hearing loss in stage T4 tumors was greater at 250 and 500 Hz but smaller at 2,000 and 8,000 Hz of PTA in comparison with smaller SDS (15). In the following year, Gerganov et al. analyzed radiological images and preoperative hearing levels of 99 SVS patients, and they adopted a volumetric method and found the hearing level was significantly correlated with the tumor stage and tumor volume (9). *Via* employing similar volumetric imaging parameters, Bathla et al. revealed that the anteroposterior, mediolateral dimensions, and the tumor volume correlated with both PTA and SDS in small SVS (mean tumor volume ~5.5 ml) (24). A similar correlation was replicated in another two volumetric analyses in smaller SVS (25, 26).

To answer whether tumor size/volume affects hearing function in SVS, the reasonable solution is performing a meta-analysis. However, various un-unified parameters regarding the hearing function and tumor size in those researches make meta-analysis difficult. Thus, we summarized the key information of the literature in **Table 1**. The methodology in tumor size/volume evaluation seems to affect the correlation outcomes. Generally, there are two ways to assess tumor size/volume in the retrieved papers, including one-dimension measurement and three-dimension volumetric analysis. In the studies adopting the traditional one-dimensional measurement or simply applying tumor grade, there were only two trend findings on the association between tumor size and hearing function (14, 15). Notably, in all four volumetric analyses, the researchers reached a positive conclusion. Apparently, in comparison with the direct measurement of the maximum diameter in one or multiple axes, the volumetric analysis of the tumor is more accurate in the assessment of tumor size. Thus, the potential relationship between the tumor size/volume and hearing function may exist in SVS, but further studies with a larger sample size and a more accurate volumetric method are needed to illuminate this hypothesis.

#### Tumor Growth

Tumor growth is another important physical feature of SVS. In a meta-analysis involving more than 4,000 patients, the average growth rate of newly diagnosed SVS is estimated to be 0.99–1.11 mm/year or 0.1–0.15 ml/year (53). The rate was even higher in a growing tumor and could be around 3 mm/year (53). However, whether tumor growth contributes to hearing deterioration is still in debate.

First of all, hearing loss could aggregate in the non-growth SVS. Graamans and his colleagues performed a retrospective investigation and analyzed the course of PTA and SDS along with the conservative treatment of non-growing tumors. They found that PTA revealed a significant increase in thresholds at almost all frequencies with a prominent decrease in speech discrimination in static SVS (34). Similar findings were reported by Patel et al. in 2015, which suggested tumor growth was not a requirement of hearing deterioration (35).

To further explore whether the hearing loss would aggregate in the growing tumors, the researchers performed the analysis in small or IAC tumors. In their rationale, tumor growth within the internal auditory canal would increase pressure on the auditory nerve and lead to hearing dysfunction. Unlike CPA tumors, the space in the IAC is extremely limited, and a tiny increase of tumor volume may result in a dramatic change of pressure. Under this logic, small SVS, especially within IAC, serve as a better subset to explore hearing loss and tumor growth. However, the current results remain controversial.

In the studies applying linear tumor growth rate, Walsh et al. and Hajioff et al. divided SVS patients into growth (>1 mm/year) and no-growth (≤1 mm/year) groups to analyze the difference in the change of mean PTA and SDS, and they found that there was a significant risk of hearing impairment in the growth tumors (27, 30). Additionally, Sakamoto et al. calculated annual tumor growth and annual hearing loss rate (based on PTA, dB/year) and recognized a correlation between the two parameters. However, they were not associated with PTA at the initial diagnosis (16). Prasad et al. further distinguished the fast growth group (≥3 mm/year) and analyzed hearing deterioration among the no-growth, slow growth (1-3 mm/year), and fast growth groups. He observed that the growing tumors tended to cause progressive hearing, but the findings were not statistically significant (28). Another study evaluated the tumor growth of 97 SVS patients and compared the relationship between the tumor growth rate and hearing outcome *via* univariate and multivariate analysis. They concluded that hearing impairment was related significantly to rapid tumor growth (≥0.10 cm^3^/year) (25). Conversely, Younes et al. used 2 mm/year to identify the growing tumor, but no significant association was spotted between tumor growth and hearing loss (29).

In the rest studies, researchers applied linear or volumetric changes of the tumor size to assess the tumor growth. In 2007, Caye-Thomasen et al. analyzed the spontaneous course of hearing in 156 patients with IAC SVS. The correlation analysis showed that the PTA deterioration rate correlated positively with the absolute growth rate (17). With longer observation, the same research group also indicated the PTA deterioration in the growing tumors was significantly higher (10). Similarly, Fayad et al. observed a significant decline of the PTA at 0.5, 1, 2, 3 kHz in the growing tumors (tumor increase >2 mm, mean PTA decrease = 28.8 dB) than those that did not (mean PTA decrease = 16.5 dB), but there was no correlation between the amount of change in hearing and the size of the tumor. Additionally, the annual hearing decreasing rate, which was calculated based on the PTA, was reported to be significantly higher in the growing SVS (31). In contrast, a retrospective case series involving 47 IAC SVS patients exhibited no significant difference in hearing loss among growing, stable, and shrinking tumors (32). In the rest two negative reports, the volumetric analysis was applied to assess the change in the tumor size accurately, but still, the correlation between hearing deterioration and tumor growth was not significant (26, 33).

#### Tumor Location

Tumor location is also a key physical property of SVS and its association with hearing loss has been discussed widely. Generally, SVS can be classified as IAC tumors when they are only intracanalicular and as CPA ones when the tumors extend extracanalicularly and locate mainly in CPA. In this review, we found four studies comparing the difference in hearing loss between IAC and CPA SVS. In a retrospective study involving 72 patients, the researchers spotted that hearing deteriorated more significantly in CPA tumors (27). Besides, Linge et al. observed that the IAC SVS patients presented a lower PTA at the frequency of 0.5, 1, 2, and 4 kHz than the tumors located in CPA (31). In comparison, Lee et al. observed that there was no significant association between hearing function (e.g., PTA, SDS, tinnitogram findings, and ABR) and tumor sites (e.g., IAC alone, IAC+CPA, IAC+CPA+brainstem compression) (21). Similarly, another prospective study involving intracanalicular and extracanalicular SVS (tumor size <10mm) demonstrated that hearing status at baseline did not correlate with the initial site of the tumor (22). Thus, the results regarding hearing loss in IAC and CPA SVS are still in debate. As we knew, few SVS originate extracanalicularly alone, which makes it difficult to compare the hearing function between the tumor originated purely in IAC and CPA. Additionally, CPA mass usually has a much larger tumor size than IAC mass, which suggests tumor size would be another confounding factor during the analysis. Thus, we should interpret the two positive reports carefully. The association between hearing function and tumor site (CPA *vs*. IAC) needs more solid evidence to support.

Generally, SVS usually arises from the glial-Schwann cell junction within IAC, and current literature fails to establish the association between the origin of tumor and hearing. For the IAC SVS, researchers proposed that the tumors could be categorized into three subgroups, including fundus tumors (no cerebrospinal fluid [CSF] between the tumor and cochlea), central tumors (CSF observed in both ends of IAC) and pours tumors (CSF only between tumor and cochlea) (17). However, there was no difference in hearing deterioration at diagnosis and observation among these three subgroups (17). Likewise, another two studies found no significant differences in hearing loss among SVS grouped by subsite in the IAC (32, 36).

#### Pressure and Tumor Filling Within IAC

Although the association between hearing and SVS subsite within IAC was weak, some research groups measured the pressure in the IAC and tried to correlate it with hearing outcomes. Badie et al. performed a pilot study and measured the preoperative intracanalicular pressure (ICaP) in 15 patients undergoing resection of SVS. Their findings suggested that ICaP directly correlated with the amount of tumor within IAC and a trendy inverse correlation between ICaP and hearing function (54). To further validate this correlation, they involved more patients and enriched the studies with preoperative auditory evoked potential (AEP) recordings. This time, the correlation between ICaP and hearing deterioration reached a significant conclusion, and they also spotted that the wave V latency of preoperative AEP recordings was associated with the change in ICaP, which suggested the pressure caused by tumor filling in the IAC might contribute to the hearing loss in SVS (55).

The measurement of ICaP is invasive and can be performed only during the surgery, which limits its application. Thus, some researchers turned to evaluate the extent of the tumor filling within IAC. This parameter directly correlates with ICaP and can be evaluated in MRI scans without invasive operation, which seems to be a promising alternative to ICaP. As noted in a recent report, Zhou et al. proposed a new MRI grading biomarker based on the percentage of tumor filling the inner auditory canal (TFIAC) to predict the hearing loss in SVS and defined the four grades accordingly with low TFIAC (<25%) in Grade I and high TFIAC in Grade IV (>75%). They found that the patients in TFIAC grade III experienced more significant hearing deterioration than Grade I patients, and TFIAC grade IV patients also had a higher rate of non-serviceable hearing (56). Besides, in another MRI study aiming to identify practical predictors of hearing loss in the conservatively managed SVS, the researchers considered the fundal cap as a candidate. It was measured as the maximal distance between the lateral most aspect of the tumor to the fundus or the lateral most aspect of the internal auditory canal, and its size was correlated with the word recognition score over time (57). It is valuable for future studies with larger sample size and longer observation to verify the association between hearing loss and tumor filling within IAC.

### Cochlear Dysfunction

Cochlear dysfunction is another leading cause of hearing loss in SVS. The cochlea is a subtle auditory organ located in the core of temporal bone. In this review, we found ten studies investigating this topic, including four reports on the structural abnormality of the cochlea (13, 37–39), three reports on the pathological changes in perilymph (40–42) and three reports regarding the functional evaluation of cochlea (43–45) (**Table 4**).

First of all, SVS may lead to structural abnormality of the cochlea and affect hearing function. Histologically, degeneration and loss of spiral ganglion cells are the prominent pathologic finding in the cochlea ipsilateral to the tumor (13, 58, 59). Autopsy analysis of 11 SVS patients indicated a significant decrease in spiral ganglion cells, loss of inner and outer hair cells, degeneration of the stria vascularis, and the spiral ligament in the tumor ear with poor hearing (13). Later, Roosli et al. confirmed these pathological findings and further detected a high rate of precipitates in the endo- and peri-lymphatic spaces (43%) with endolymphatic hydrops in 25% of SVS patients (37). However, due to the limitation of the autopsy samples, the correlation analysis between the cochlea degeneration and hearing loss rarely works. Recently, endolymphatic hydrops, which could be detected in MRI scans, was correlated with poor hearing in SVS (60). Eliezer et al. analyzed T2-weighted MRI scans in 23 patients with obstructive SVS, which revealed a moderate correlation between hearing loss and the volume of the vestibular endolymphatic space and utricle (38). Using the same MRI sequence, another research group performed the study in 183 SVS patients and noticed that saccular dilation was common in SVS (28% unilaterally and 15.7% bilaterally), and a significant association was found between saccular dilation and progressive sensorineural hearing loss (39).

Other than the structural abnormality of the cochlea, several studies observed elevated levels of protein in the perilymph of SVS, which exhibited a high fluid-attenuated inversion recovery (FLAIR) signal or a decreased signal on T2-weighted MRI scans (60–62). In one small cohort study involving 28 SVS patients, Yamazaki et al. calculated the signal intensity ratio between the affected cochlea and medullar on the FLAIR images (CM ratio). Notably, the CM ratio was higher in the tumor ear, and this elevation was positively correlated with the degree of hearing loss (40). Later, a similar correlation was replicated in a study with a larger sample size in the IAC tumors (42). In contrast, another MRI investigation regarding 3D FLAIR imaging in SVS failed to exhibit the correlation between hearing function and intensity ratio of the labyrinth in the tumor ears, despite significant signal changes observed in the cochlea (41). Thus, further investigations are needed to validate the association between hearing levels and the change of perilymph in SVS patients.

In the functional assessment of cochlea, there is evidence that supports the cochlear origin of hearing loss in SVS. In a study applying the distortion products of otoacoustic emissions (DPOAEs), DPOAEs amplitudes in the tumor ear were decreased compared with the contralateral ear at multiple frequencies in SVS patients with early hearing loss (43). The DPOAEs are evoked OAEs, which is to test the functional integrity of the cochlea’s outer hair cells. Alterations in the test suggested the cochlea was involved in SVS inducing hearing loss. With more participants, Ferri et al. classified the SVS patients according to DPOAEs and auditory measurement. In most SVS (~75%), DPOAEs were “compatible” with hearing levels (44). Additionally, Byun et al. applied non-invasive threshold-equalizing noise (TEN) tests to evaluate the cochlear dead region, and more non-functional regions were detected in the tumor ear, which was associated with hearing loss (45).

### Impairment of Auditory Pathway and Cortex

Besides the direct mechanical insults caused by tumors and secondary cochlear dysfunction, the rest sections of auditory pathway in brain can also be affected by SVS. Based on diffusion-weighted MR imaging, Rueckriegel et al. first visualized the different sections of the auditory pathway between the inferior colliculus and the auditory cortex in SVS patients by applying probabilistic tractography. Moreover, they observed a significant volume decrease in the lateral and diencephalic sections of the auditory pathway on the contralateral hemisphere (63). In the same year, another group of researchers performed voxel-based morphometry to evaluate the volume changes of gray matter in 42 SVS patients compared with 24 healthy controls (64). They observed that the gray matter volume of SVS patients increased significantly in the somatosensory and motor systems while decreased in the auditory and visual cortex. Notably, the GM volume decreases in the primary auditory cortex, including the superior temporal gyrus and Heschl’s gyrus, and it correlated with hearing impairment, which suggested SVS could have a profound effect on brain plasticity and contributed to hearing loss (64).

### Molecular and Genetic Changes

However, some patients develop audiometric threshold shifts despite the lack of tumor growth. Besides, Stankovic et al. found that patients with abnormal baseline in the ipsilateral hearing demonstrated a higher likelihood of developing moderate hearing loss in the ears contralateral to the lesion (48). Therefore, additional intrinsic biological differences should exist among SVS patients, which leads to different degrees of hearing dysfunction. Recently, several pathogenic mechanisms have been identified to contribute to SVS-associated hearing loss, including genetic alteration, growth factors, inflammatory response, *etc*. (**Table 5**).

#### NF2 and Other Generic Alteration

In 1993, the NF2 gene was first identified in the patient with neurofibromatosis type 2 and was regarded as the classic gene involving the tumorigenesis of SVS (65). To date, more than 200 genetic alterations have been found in the NF2 gene, including single-base substitutions, insertions, missense, deletions, DNA Methylation, *etc*. (66). The product of the NF2 gene is merlin, which is known for its tumor-suppressing properties. Physiologically, merlin can bind to DCAF1 and suppress cell proliferation *via* inhibiting E3 ubiquitin ligase CRL4 (67). In the majority of SVS, merlin is inactivated and favors tumor development. Interestingly, one study found that NF2 gene mutation was associated with hearing loss in SVS patients (11). In this research, Lassaletta et al. analyzed DNA in 51 surgical samples after the removal of unilateral SVS, and they observed that the NF2 mutations had lower mean corrected PTA thresholds compared with those without the mutations, which was thought to be due to NF2-related growth pattern (11). However, the relationship between the NF2 gene and the hearing function in SVS still needs more verification.

Besides the NF2 gene, another six genes, including TP73, PEX5L, RAD54B, PSMAL, CEA, and CCND1, were associated with hearing deterioration in SVS (46–48). Lassaletta et al. investigated the methylation status of 16 tumor-related genes in SVS patients, and the incidence of TP73 methylation was 9%. Compared with the unmethylated patients, the corrected PTA was significantly higher in the methylated patients (43 dB *vs*. 19 dB, p=0.04) (46). Additionally, they also exhibited that Cyclin D1 was expressed in more than half of SVS, and the negative Cyclin D1 expression was associated with a longer duration of deafness and higher 2,000-Hz hearing PTA thresholds (47). In another study regarding the whole-genome expression profiling of SVS, elevated expression of CEA and decreased expression of PEX5L, PSMAL, and RAD54B were associated with poor hearing (48). Together with the potential link between CEA and peroxisome, the altered expression of PEX5L, which was thought to modulate the import of peroxisomal protein, suggested the peroxisomal dysfunction might contribute to hearing loss in SVS. The other two genes (RAD54B and PSMAL) may have indirect roles and cause the inner ear degeneration (48).

#### Inflammatory Response

Previous studies suggested the maladaptive activation of inflammation contributed to the SVS pathogenesis. Specifically, in the histological examination, prominent immune infiltration could be observed in the majority of SVS, which involved microglia/macrophage, lymphocytes, neutrophils, etc. (68) Further, cyclooxygenase 2, a key enzyme participating in prostaglandin synthesis and major modulator of the inflammation, is strongly expressed in the SVS tissue specimen and was associated with tumor proliferation rate (69). However, the association between inflammation and hearing deterioration has been rarely explored, and there are only two papers regarding this topic. In the ex vivo model of murine cochlear explant, the extent of cochlear explant damage caused by tumor secretions correlated with the corresponding hearing function in the SVS subjects. Further analysis of tumor secretions identified tumor necrosis factor-alpha (TNFα) as the ototoxic molecule, and its neutralization in SVS secretions could partially reverse hair cell loss, which highlighted the role of TNFα in the cochlear origin of hearing loss (49). Recently, Stankovic et al. exhibited the over-expression of multiple critical genes associated with the inflammasome in SVS, and the two associated proteins (NLRP3 and IL-1ß) were preferentially present in tumors with poor hearing (12).

#### Growth Factors and Other Secreted Vesicles

Other than inflammatory cytokines, growth factors and other tumor-associated secretions have been identified in SVS-associated hearing loss. Among growth factors, platelet derived growth factor alpha (PDGFA), and fibroblast growth factor 2 (FGF2) were reported to be associated with hearing levels among SVS (49, 50, 70). In a correlation study between cDNA microarray expression and SVS clinical features, PDGFA, originally identified as mitogenic factors for smooth muscle cells, was inversely correlated with hearing loss (70). Dilwali et al. performed a comparative screening of molecular biomarkers in SVS and observed that SVS with good hearing secretes a higher level of FGF2 (50). Furthermore they validated the otoprotective role of FGF2 in cochlear explants (49). In addition, vascular endothelial growth factor (VEGF) was another potential factor associated with poor hearing in SVS. In neurofibromatosis type 2, VEGF was elevated and reported to contribute to tumor growth and hearing loss (71). In clinical and experimental research, anti-VEGF treatment could improve hearing function *via* normalizing the tumor vasculature, improving vessel perfusion, and delivery of oxygenation (71, 72). In sporadic SVS, the majority of tumors expressed VEGF and its receptors, which correlated with tumor growth, disease recurrence, and preoperative irradiation (73). However, there is no study focused on the association between hearing and VEGF, which needs to investigate in future studies.

Additionally, extracellular vesicles and matrix metalloprotease 14 (MMP-14) in tumor-associated secretions were found to induce hearing loss in SVS. Soares et al. observed that Human VS cells could secret extracellular vesicles and transfer tumor-derived RNAs to cochlear cells. These extracellular vesicles from VS associated with poor hearing induced the apoptosis of spiral ganglion cells and destroyed the cultured cochlear explants (51). Recently the expression and activity of MMP-14 in the plasma and tumor secretions were observed to be correlated with the degree of hearing loss in VS. Moreover, in an ex vivo model of cochlear explant cultures, MMP-14 induced the damage of spiral ganglion neuronal fibers and synapses at physiologic concentrations (52).

### Current Treatment Options

The classic treatments for SVS are microsurgery and radiotherapy, which can introduce direct injury to the cochlea nerves and lead to a profound ipsilateral hearing decrease. To minimize the iatrogenic hearing impairment and facilitate hearing rehabilitation, new techniques (such as detailed presurgical planning, neuromonitoring of the cochlear nerve, cochlear implantation, fractionated stereotactic radiotherapy, *etc*.) are proposed to favor hearing preservation (7, 74–77). While the intraoperative monitoring of facial nerve has become a standard technique in the surgery of SVS, some clinicians tried intraoperative brainstem auditory evoked response and cochlear compound action potentials to identify the location of cochlear nerves and monitor its functional status (76, 78). Current literature supports its therapeutic value on hearing preservation in small-medium-sized tumors, but its role in the large SVS remains to be determined (76, 77, 79). Cochlear implants (CI), as the most successful neural prosthesis, also play an important role in the hearing rehabilitation for SVS patients after surgery. CI can be placed either simultaneously with the resection of SVS or in a delayed manner, but the timing of this procedure seems not to influence overall hearing outcomes (80–82). A recent systematic including 29 studies with 93 patients indicated that servable hearing with CI is feasible after VS surgery if the cochlear nerve is anatomically preserved (82). Among those patients, 83.9% used the CI device daily and 54.8% achieved open-set speech (82).

For the SVS patients in the observation, the treatment options for hearing loss are still limited. Carlson et al. implanted conventional CI arrays in nine patients with intracochlear and intralabyrinthine VS, and seven patients achieved good open-set word recognition after 21 months of clinical follow-up. However, in this study, the authors intentionally left VS *in situ* to ensure the integrity of the cochlear nerve, which caused additional concerns about tumor progression and limited its clinical applications (83). Thus, despite that CI may provide new options for hearing restoration in SVS, it can only serve as a backup if the patients have no other options (84). In contrast, there is emerging evidence that supports the otoprotective effect of Bevacizumab (BEV). As an inhibitor of the VEGF pathway, BEV was initially reported to improve hearing in patients with NF2 and contribute to tumor shrinkage at the same time (71, 85). In a recent case report, Karajannis et al. administrated a low dose of Bevacizumab (2.5 mg/kg every 4 weeks) in an adult female patient with progressive SVS (86). Notably, after continuous therapy for 33 months, the tumor exhibited a 67.8% shrinkage in comparison with the pretreatment baseline and the patient maintained normal hearing in the ipsilateral ear (86). Therefore, BEV may be a novel and effective treatment alternative for SVS patients with hearing loss, and it would be valuable for future research to validate its therapeutic effect.

## Conclusions

Overall, multiple factors seem to be associated with hearing impairment in SVS. The growth pattern of tumor, cochlear dysfunction, impairment of auditory pathway and cortex, genetic and molecular alteration contribute to hearing deterioration. First of all, the growth pattern of the tumor may play a role in the pathogenesis of hearing loss. Larger tumor size, faster tumor growth, and more tumor extension within IAC possibly aggregate the mechanical compression of the cochlear nerve and brain stem, which impairs the transmission and processing of auditory signals. Secondly, SVS may also result in cochlear damage, characterized by structural abnormality and high protein levels in the perilymph, which subsequently have a negative influence on the pickup of auditory stimulus and generation of nervous impulses. Thirdly, the growth pattern of SVS, cochlear dysfunction, and long-term hearing dysfunction may impair and reframe the auditory pathway and cortex, which aggregate hearing loss. Fourthly, genetic alteration in SVS (e.g., DNA mutations, abnormal methylation, *etc*.) can lead to dysregulation of multiple genes (e.g., NF2, TP73, *etc*.), which promote cell proliferation, impair DNA repair, cause peroxisomal dysfunction, *etc*. Besides, the tumor cells in SVS can secret extracellular vesicles, proinflammatory cytokines, and growth factors, which exert harmful effects *via* paracrine pathways in both cochlea and brain.

**Figure 2:**
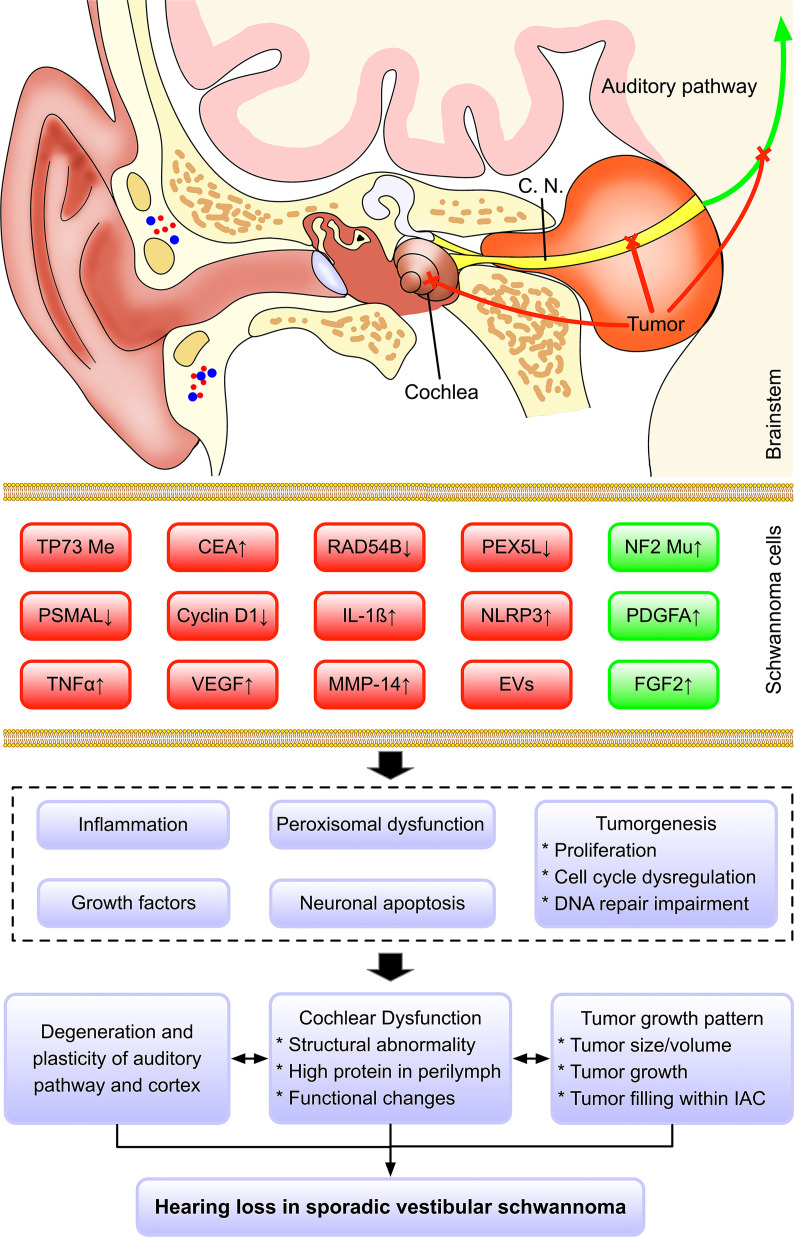
Hypothesized mechanism regarding hearing loss in sporadic vestibular schwannoma. Multiple factors may contribute to the hearing impairment in SVS, including the growth pattern of tumor, cochlear dysfunction, impairment of auditory pathway and cortex, genetic and molecular changes. Based on our findings, we proposed a multi-level hypothesis that genetic and molecular changes in SVS might influence the various cellular activity (e.g., cell proliferation, peroxisomal dysfunction, DNA repair, angiogenesis, *etc*.) and subsequently promote the secretion of ototoxic factor and tumorigenesis with distinct tumor growth pattern, which might impair the auditory related structures (e.g., cochlea, cochlear nerves, auditory pathway, cortex, etc.) *via* either directly mechanical compression or cytotoxicity *via a* paracrine pathway. Color code: red, the factors contribute to hearing loss; green, the factors are associated with good hearing.

Based on our findings, we proposed multi-level hypotheses that genetical and molecular changes in SVS might influence the various cellular activity (e.g., cell proliferation, peroxisomal dysfunction, DNA repair, angiogenesis, *etc*.) and subsequently promote the secretion of ototoxic factor and tumorigenesis with distinct tumor growth pattern, which might impair the auditory related structures (e.g., cochlea, cochlear nerves, auditory pathway, cortex, *etc*.) *via* either directly mechanical compression or cytotoxicity *via* a paracrine pathway (**Figure 2**). However, our current understanding of this topic is still limited, and future clinical and experimental studies should further test this multifactorial hypothesis and dig deeper into its underlying mechanism.

## Data Availability Statement

The original contributions presented in the study are included in the article/**Supplementary Material**. Further inquiries can be directed to the corresponding authors.

## Author Contributions

LW and HZ supervised and conceived the study, analyzed and interpreted data, and wrote and revised the manuscript. JG and YZ performed the study, searched the literature, collected and analyzed data, and wrote the first draft of the manuscript. JW searched the literature, collected and analyzed data. DL and FZ conceived the study and contributed to data interpretation and substantial revision of the manuscript. All authors contributed to the article and approved the submitted version.

## Supplementary Material

The Supplementary Material for this article can be found online at: https://www.frontiersin.org/articles/10.3389/fonc.2021.687201/full#supplementary-material

## Conflict of Interest

The authors declare that the research was conducted in the absence of any commercial or financial relationships that could be construed as a potential conflict of interest.

## Publisher’s Note

All claims expressed in this article are solely those of the authors and do not necessarily represent those of their affiliated organizations, or those of the publisher, the editors and the reviewers. Any product that may be evaluated in this article, or claim that may be made by its manufacturer, is not guaranteed or endorsed by the publisher.

## Abbreviations

3D, three dimensional; ABR, auditory brainstem response; AEP, auditory evoked potential; BEV, bevacizumab; CEA, carcinoembryonic antigen; CI, cochlear implants; CM ratio, signal intensity ratio between the affected cochlea and medullar on the FLAIR images; CPA, cerebellopontine angle; DNA, deoxyribonucleic acid; DPOAEs, distortion products of otoacoustic emissions; DRs, dead regions; FGF2, fibroblast growth factor 2; FLAIR, fluid-attenuated inversion recovery; IAC, internal auditory canal; ICaP, intracanalicular pressure; IL-1ß, Interleukin-1ß; MMP-14, matrix metalloprotease 14; MRI, magnetic resonance imaging; NLRP3, NLR family pyrin domain containing 3; PDGFA, platelet derived growth factor alpha; PEX5L, peroxisomal biogenesis factor 5-like; PSMAL, prostate-specific membrane antigen-like; PTA, pure tone average; RAD54B, RAD54 homolog B; RNA, ribonucleic acid; SDS, Speech discrimination score; SRT, speech reception thresholds; SVS, sporadic vestibular schwannoma; TEN tests, the threshold-equalizing noise test; TFIAC, tumor filling the inner auditory canal; TNFα, Tumor necrosis factor alpha; VEGF, vascular endothelial growth factor; VS, vestibular schwannoma.
