# Supplementary material for: Current Understanding of Hearing Loss in Sporadic Vestibular Schwannomas: A Systematic Review
Source: Front Oncol. 2021 Aug 12;11:687201. doi: 10.3389/fonc.2021.687201 (PMC8406761; doi:10.3389/fonc.2021.687201)
Supplement: Supplementary file 1 [file DataSheet_1.docx]

Supplement materials: Detailed Search Strategy

- Limitation for all databases, 1. publication from Jan 2000 to Dec 2020; 2. English literature.
- Search Criteria for PubMed using Medical Subject Headings (MeSH)

1. Vestibular schwannoma
2. Acoustic neuroma
3. 1 OR 2
4. Hearing loss
5. Hearing Impairment
6. Hypoacusis
7. Transitory Deafness
8. 4 OR 5 OR 6 OR 7
9. 3 AND 8

- Search Criteria for Ovid: Medline using Medical Subject Headings (MeSH)

1. Vestibular schwannoma/

2. Vestibular schwannoma.mp.

3. Acoustic Neuroma/

4. Acoustic Neuroma.mp.

5. 1 OR 2 OR 3 OR 4

6. Hearing Loss/

7. Hearing Loss.mp.

8. Hypoacusis/

9. Hypoacusis.mp.

10. Hearing Impairment/

11. Hearing Impairment.mp.

12. Transitory Deafness/

13. Transitory Deafness.mp.

14. 6 OR 7 OR 8 OR 9 OR 10 OR 11 OR 12 OR 13

15. 5 AND 14

- Similar search criteria for EMBASE database using Focused Medical Subject Headings (MeSH)
- Search Criteria for Web of Science using Medical Subject Headings (MeSH)

1. Vestibular schwannoma
2. Acoustic neuroma
3. 1 OR 2
4. Hearing loss
5. Hearing Impairment
6. Hypoacusis
7. Transitory Deafness
8. 4 OR 5 OR 6 OR 7
9. 3 AND 8
